# Clinical effectiveness and safety of spinal anaesthesia compared with general anaesthesia in patients undergoing hip fracture surgery using a consensus-based core outcome set and patient-and public-informed outcomes: a systematic review and meta-analysis of randomised controlled trials

**DOI:** 10.1016/j.bja.2022.07.031

**Published:** 2022-09-28

**Authors:** Setor K. Kunutsor, Pravakar B. Hamal, Sara Tomassini, Joyce Yeung, Michael R. Whitehouse, Gulraj S. Matharu

**Affiliations:** 1 National Institute for Health Research Bristol Biomedical Research Centre, University Hospitals Bristol and Weston NHS Foundation Trust and University of Bristol, Bristol, UK; 2 Musculoskeletal Research Unit, Translational Health Sciences, Bristol Medical School, University of Bristol, Learning & Research Building (Level 1), Southmead Hospital, Bristol, UK; 3 Warwick Clinical Trials Unit, University of Warwick, Warwick, UK; 4 University Hospitals of Birmingham NHS Foundation Trust, Birmingham, UK

**Keywords:** complication, core outcome set, general anaesthesia, hip fracture, meta-analysis, mortality, spinal anaesthesia, systematic review

## Abstract

**Background:**

We conducted a systematic review and meta-analysis of contemporary RCTs to determine the clinical effectiveness of spinal *vs* general anaesthesia (SA *vs* GA) in patients undergoing hip fracture surgery using a consensus-based core outcome set, and outcomes defined as important by patient and public involvement (PPI) initiatives.

**Methods:**

RCTs comparing any of the core outcomes (mortality, time from injury to surgery, acute coronary syndrome, hypotension, acute kidney injury, delirium, pneumonia, orthogeriatric input, being out of bed at day 1 postoperatively, and pain) or PPI-defined outcomes (return to preoperative residence, quality of life, and mobility status) between SA and GA were identified from MEDLINE, Embase, Cochrane Library, and Web of Science (2000 to February 2022). Pooled relative risks (RRs) and mean differences (95% confidence intervals [CIs]) were estimated.

**Results:**

There was no significant difference in the risk of delirium comparing SA *vs* GA (RR=1.07; 95% CI, 0.90–1.29). Comparing SA *vs* GA, the RR for mortality was 0.56 (95% CI, 0.22–1.44) in-hospital, 1.07 (95% CI, 0.52–2.23) at 30 days, and 1.08 (95% CI, 0.55–2.12) at 90 days. Spinal anaesthesia reduced the risk of acute kidney injury compared with GA: RR=0.59 (95% CI, 0.39–0.89). There were no significant differences in the risk of other outcomes. Few studies reported PPI-defined outcomes, with most studies reporting on one to three core outcomes.

**Conclusions:**

Except for acute kidney injury, there were no differences between SA and GA in hip fracture surgery when using a consensus-based core outcome set and patient and public involvement-defined outcomes. Most studies reported limited outcomes from the core outcome set, and few reported outcomes important to patients, which should be considered when designing future RCTs.

**PROSPERO registration:**

CRD42021275206

**Editor's key points**

• The optimal anaesthesia technique (spinal *vs* general) in hip fracture surgery is controversial, with little robust evidence for one method over others.
• There is wide variability in outcome definitions and reporting among studies, which affects interpretation of previous studies and meta-analyses.
• In this systematic review, the authors identified that most outcomes were not affected by spinal *vs* general anaesthesia.
• Most studies reported few outcomes from the consensus-based core outcome set and only a few studies reported on outcomes important to patients. Such outcomes should be incorporated into future studies.

Hip fractures are devastating injuries, constituting a global public health burden and remain one of the largest healthcare challenges of the 21st century. The incidence increases with advancing age^1^ and the number of hip fractures is expected to increase to 6.26 million per year in 2050.^2^ Hip fractures impact on patient's psychological, functional, and social wellbeing, and account for substantial healthcare system costs.^3^^,^^4^ In 2017, hip fractures cost the NHS £1 billion,^5^ which is projected to increase to £5.6 billion in 2033.^6^ Patients with hip fractures have a significant risk of mortality; despite optimal care, mortality rates at 30 days and 1 yr are 10% and 25%, respectively.^7^

Almost all patients with hip fracture undergo surgery, requiring either neuraxial or general anaesthesia (GA).^5^ Given the risk profile of hip fracture patients (older age, frailty comorbidities such as cardiac and respiratory diseases), surgery is associated with a high risk of developing postoperative complications including delirium, myocardial infarction (MI), pneumonia, stroke, and mortality.^8^^,^^9^ The type of anaesthesia may influence the outcome but the ideal regimen has not been identified with inconsistent findings reported in RCTs.^10^^,^^11^ Several meta-analyses have attempted to aggregate the findings from individual RCTs, but with inconclusive results.^12^, ^13^, ^14^, ^15^ Limitations of previous reviews could have limited the validity of their findings; these included (1) pooled analysis of very few trials, hence reducing power to effectively make head-to-head comparisons^16^^,^^17^; (2) inclusion of studies that employed anaesthetic drugs no longer used in clinical practice^12^; and (3) restrictive outcome selection.^16^ The National Institute for Health and Care Excellence (NICE) recommend further RCTs to assess the effect of anaesthesia on outcomes after hip fracture surgery.^18^

It has been suggested that the failure of RCTs and meta-analyses to date to identify the optimal anaesthesia type for hip fracture patients, may partly be attributable to wide variability in outcome definitions and reporting among studies.^19^ There are several core outcome sets (COSs) that have been proposed for perioperative research. The Oxford core outcome set^20^ was developed in 2014 and designed to be used in interventional studies investigating patients with a hip fracture and includes a set of patient-reported outcomes such as pain and activity of daily living (ADL) and clinical outcomes such as mortality. The Standardised Endpoints and COMs for Perioperative and Anaesthetic Care (Step-COMPAC) initiative aims to produce a core outcomes set for different aspects of perioperative care (patient comfort, clinical indicators, cognition and stroke, cardiovascular, respiratory, renal, bleeding and transfusion, organ failure and survival, cancer and long-term survival, patient-centred outcomes).^21^ The Step-COMPAC outcomes are suitable for perioperative clinical trials but did not include outcomes that are most relevant to specific targeted patient populations.

To enable more robust comparisons of anaesthetic techniques, O'Donnell and colleagues^19^ have recently developed a consensus-based set of 10 core outcomes for use in all future perioperative RCTs evaluating the effects of anaesthesia in hip fracture patients. This COS was developed during a comprehensive consensus process consisting of a systematic review of the literature, three rounds of a Delphi survey, two consensus webinars, and face-to-face patient meetings.^19^ The COS provides an updated list of patient focused outcomes including mortality, time from injury to surgery, acute coronary syndrome, hypotension, acute kidney injury (AKI), delirium, pneumonia, orthogeriatric input, being out of bed at day 1 postoperatively, and pain. Key process outcomes (e.g. time to surgery, orthogeriatrics input) which may impact patient care and recovery after hip fracture have also been included.

Recent patient and public involvement (PPI) initiatives for hip fracture and qualitative systematic reviews have identified additional important outcomes such as return to preoperative residence (i.e. home, residential care, nursing home), quality of life (QOL), and mobility status.^20^^,^^22^ Since the last relevant reviews on the topic,^12^^,^^15^ several new RCTs have been published^10^^,^^23^, ^24^, ^25^ including two large multicentre RCTs namely the Regional Anesthesia *vs* General Anesthesia (RAGA)^24^ and the Regional *vs* General Anesthesia for Promoting Independence after Hip Fracture (REGAIN) trials.^25^

We conducted a systematic review and meta-analysis of all contemporary RCTs to determine the clinical effectiveness of spinal anaesthesia (SA) compared with GA in patients undergoing hip fracture surgery using the O'Donnell COS and outcomes defined as important by PPI initiatives.

## Methods

### Data sources and search strategy

This review was conducted using Preferred Reporting Items for Systematic Reviews and Meta-Analyses (PRISMA) guidelines^26^ (Supplementary material 1) and it was based on a pre-defined protocol registered in the prospective register of systematic reviews, PROSPERO (CRD42021275206). To find studies eligible for inclusion, we searched the literature databases MEDLINE, Embase, and The Cochrane library from year 2000 to October 7, 2021, without language restrictions. The search was updated to February 10, 2022 to identify any additional studies published during the review process. The computer-based searches used a combination of keywords or terms relating to the population (e.g. ‘hip fracture’, ‘femoral fracture’, ‘femoral neck fracture’) and intervention/comparator (e.g. ‘general anesthesia’, ‘regional anesthesia’, ‘spinal anesthesia’, ‘neuraxial anesthesia’, anaesthesia), with full details of the search strategy provided (Supplementary material 2). Titles and abstracts of retrieved citations were initially independently screened by two authors (PBH and ST) to assess suitability for potential inclusion. Screening was conducted using Rayyan (Rayyan Systems Inc., Cambridge, MA, USA; http://rayyan.qcri.org).^27^ The titles and abstracts of the retrieved citations were uploaded to Rayyan. Reviewers then screened the studies based on titles, abstracts, and/or predefined lists of keywords that were most likely to hint to either include or exclude a study. This was followed by full-text evaluation, which was independently conducted by two authors (PBH and ST). Disagreements regarding eligibility of an article/study were discussed and resolved by mutual agreement with involvement of four other authors (SKK, JY, MRW, and GSM). The reference lists of relevant studies and review articles were manually scanned for additional studies and citing references were also checked in Web of Science.

### Eligibility criteria

Studies were eligible if they were RCTs that: (1) compared SA with GA in patients undergoing hip fracture (defined as fracture of the proximal femur) surgery and (2) reported on any of the 10 consensus-based core outcomes,^19^ any outcome defined as important by PPI initiatives, or both.^28^, ^29^, ^30^, ^31^ Studies that used sedation, peripheral nerve blocks, or both in conjunction with SA or GA were included. The following were excluded: (1) non-randomised studies; (2) studies of anaesthetic technique or drugs not considered contemporary standard practice (e.g. halothane, enflurane, xenon); and (3) studies of patients undergoing hip fracture surgery alongside other surgery or in the context of polytrauma.

### Outcomes evaluated

Outcomes evaluated included the set of 10 core outcomes (mortality, time from injury to surgery, acute coronary syndrome, hypotension, AKI, delirium, pneumonia, orthogeriatric input, being out of bed at day 1 postoperatively, and pain).^19^ These are recommended for use in all future perioperative RCTs evaluating the effects of anaesthesia after hip fracture surgery. Additional outcomes evaluated included return to preoperative residence (i.e. home, residential care, nursing home), QOL, and mobility status; these have been defined as equally important by recent PPI initiatives for hip fracture patients.^20^^,^^22^ For all the outcomes assessed, we accepted the range of definitions as reported by the included studies.

### Data extraction and assessment of risk of bias and quality of evidence

One author (ST) initially extracted information from the eligible RCTs into a predesigned data collection spreadsheet. A second author (SKK) independently extracted data from a random sample of ∼20% and checked the extracted data for accuracy using the original articles. Data were extracted on study publication date, geographical location, patient characteristics (baseline age, sex), study design characteristics (randomisation, allocation concealment, blinding, duration), type of surgery and anaesthesia, intervention and comparator groups, use of nerve blocks, sedation, or both in conjunction with anaesthesia, outcomes of interest and their counts or means with standard deviations, and risk estimates with their 95% confidence intervals (CIs). Data for one of the studies which was reported in Japanese^32^ was extracted from a previously published Cochrane review^12^ because of challenges with translation. A number of investigators were contacted to provide additional data, but none responded to our requests. The Cochrane Collaboration's risk of bias tool was used to assess the risk of bias of included RCTs.^33^ This tool evaluates seven possible sources of bias, which are random sequence generation, allocation concealment, blinding of participants and personnel, blinding of outcome assessment, incomplete outcome data, selective reporting, and other bias. For each individual component, studies were classified into low, unclear, and high risk of bias. We also used the Grading of Recommendations Assessment, Development and Evaluation (GRADE) tool to assess the quality of the body of evidence, based on study limitations, inconsistency of effect, imprecision, indirectness, and publication bias.^34^

### Statistical analyses

Summary measures of effect were presented as relative risks (RRs) (95% CIs) for binary outcomes and mean differences (95% CIs) for continuous outcomes. For the majority of studies that did not report RR estimates, these were estimated from the extracted raw counts for the intervention and comparator. For data reported as medians, ranges, and 95% CIs, means and standard deviations were calculated as described by Hozo and colleagues.^35^ Measures of effect were pooled using random-effects models to minimise the effect of heterogeneity.^36^ Where appropriate, fixed-effects models were used in parallel analyses. Standard χ^2^ tests and the *I*^2^ statistic were used to quantify the extent of statistical heterogeneity across studies.^37^^,^^38^ For the outcome of delirium (*n*=9 studies), stratified analyses according to pre-specified study-level characteristics were conducted using random effects meta-regression.^39^ We planned to assess for small study effects using formal tests such as Begg's funnel plots^40^ and Egger's regression symmetry test^41^; however, these were not done because of the limited number of studies (<10) for each outcome assessed.^42^ Given that the RAGA trial included a spinal, epidural, or combined spinal and epidural arm,^24^ we conducted sensitivity analyses which involved excluding the RAGA trial for all outcomes that RAGA contributed to and we re-analysed the data to ensure robustness of the results. All analyses were conducted using Stata version MP 17 (Stata Corp, College Station, TX, USA). For outcomes that could not be pooled, the results were summarised narratively.

## Results

### Study identification and selection

**Fig 1:**
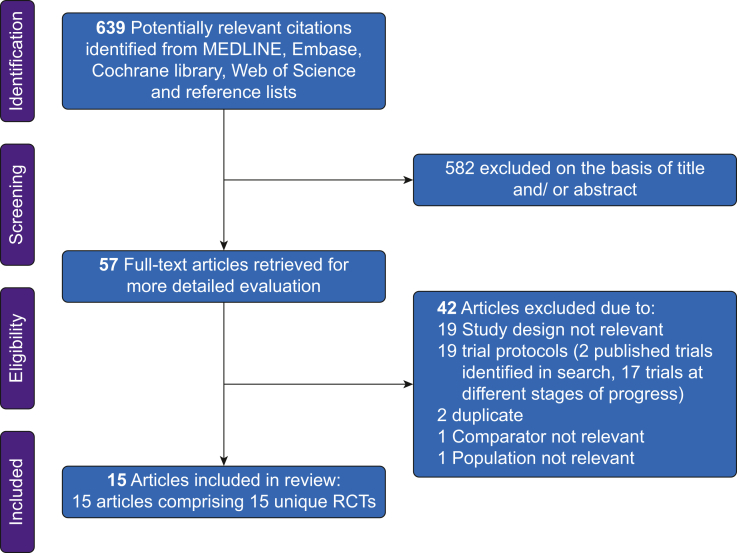
PRISMA flow diagram. PRISMA, Preferred Reporting Items for Systematic Reviews and Meta-Analyses.

Database searches and manual screening of references lists identified 639 potentially eligible articles. After initial title and abstract screening, 57 underwent full text evaluation. After detailed evaluation, 42 articles were excluded. Fifteen unique RCTs published between 2003 and 2022 were finally included in the review (Fig 1).^10^^,^^23^, ^24^, ^25^^,^^32^^,^^43^, ^44^, ^45^, ^46^, ^47^, ^48^, ^49^, ^50^, ^51^, ^52^

### Study characteristics and risk of bias

Relevant baseline characteristics of each RCT including description of anaesthetic regimens are summarised (Table 1). The 15 RCTs involved 3866 patients with hip fractures (SA=1874 and GA=1992). All trials randomised patients to receive either SA or GA except for the RAGA trial, which randomised patients to receive either regional anaesthesia (including spinal [20.2%], epidural [6.4%], or both techniques combined [73.4%]) or GA.^24^ Overall, seven studies were conducted in Asia (China, Iran, Israel, Japan, and South Korea), six in Europe (France, Greece, Italy, and UK), and two in North America (USA). The mean baseline age ranged from 66.1 to 86.0 yr, with a weighted mean of 77.9 yr. Follow-up time ranged from 10 min to 12 months postoperatively. Using the Cochrane Risk of Bias tool, seven trials demonstrated a low risk of bias in random sequence generation and four trials demonstrated a low risk of bias in allocation concealment. Twelve trials demonstrated a high risk of bias in one or more domains (Supplementary material 3).

**Table 1:** Baseline characteristics of included trials. ∗Age range not reported. GA, general anaesthesia; NR, not reported; SA, spinal anaesthesia.

First author, year of publication	Country	Population	Baseline year of recruitment	Age range (yr)	Males (%)	Follow-up time	Spinal anaesthesia	General anaesthesia	No. randomised	No. in SA group	No. in GA group
Kamitani,^32^ 2003	Japan	Femoral neck fracture	NR	82∗	10.0	4 days	0.5% isobaric bupivacaine	Propofol (0.5–1 mg), vecuronium (0.5–1 mg kg^−1^), nitrous oxide, sevoflurane, and fentanyl (0.1–0.2 mg kg^−1^)	40	19	21
Casati,^44^ 2003	Italy	Femur fracture	2003	>65	6.6	7 days	Hyperbaric bupivacaine 0.5% 7.5 mg	Sevoflurane anaesthesia	30	15	15
Hoppenstein, 2005^46^	Israel	Femoral neck fracture	2005	≥60	NR	Intraoperative period and 10 min postoperatively	Isobaric bupivacaine 4 mg with fentanyl 25 μg	Induction with i.v. fentanyl and thiopental, maintained with isoflurane and 70% nitrous oxide with vecuronium for muscle relaxation	60	30	30
Biboulet,^43^ 2012	France	Hip fracture	2012	>75	27.9	30 days	Bupivacaine 2.5 mg as loading dose with 2.5 mg top-ups if sensory level decreased below T12 with additional femoral nerve block (30 ml ropivacaine 5%)	Propofol continuous infusion or sevoflurane with fentanyl for analgesia and femoral nerve block with 5% ropivacaine (30 ml)	45	15	30
Messina,^47^ 2013	Italy	Hip fracture	2008–9	>75	35.0	Intraoperative period	Levobupivacaine 7.5 mg plus sufentanil 5 μg	Propofol, sevoflurane, remifentanil and cisatracurium	20	10	10
Parker,^49^ 2015	UK	Acute hip fracture	2007–12	>49	27.0	1 yr	NR	NR	322	158	164
Neuman,^48^ 2016	USA	Femoral neck and pertrochanteric fractures	2014–5	≥18	75.0	5 days	Spinal with i.v. sedation	NR	12	6	6
Haghighi,^45^ 2017	Iran	Acute hip fracture	2016	>60	80.0	Intraoperative and immediate postoperative period only	1.5 ml of 5% lidocaine with epinephrine	Sevoflurane anaesthesia.	100	50	50
Tzimas,^50^ 2018	Greece	Femur fracture	2015–7	>65	47.1	30 days	Fentanyl 20 5 μg and ropivacaine 0.75%	Fentanyl 3–5 mg kg^−1^ and propofol 1.5 mg kg^−1^. Desflurane used to maintain anaesthesia	70	37	33
Meuret,^51^ 2018	France	Hip fracture	2006–7	>75	20.0	30 days	Isobaric bupivacaine 5 mg with sufentanil 5 μg (single injection)	Induction with i.v. remifentanil and etomidate, maintenance with remifental and desflurane titrated to BIS	40	19	21
Shin,^10^ 2020	South Korea	Hip fracture	2015–9	>65	26.1	90 days	0.5% bupivacaine	Volatile anaesthesia with desflurane or TIVA with propofol infusion	186	62	124
Neuman,^25^ 2021	USA	Femoral neck, intertrochanteric or subtrochanteric fracture	2016–21	≥50	33.0	60 days	Single-injection spinal block with sedation. Crossover to GA permitted. At discretion of anaesthetist	Volatile anaesthetic at discretion of treating team	1600	795	805
Ren,^52^ 2021	China	Proximal femoral fracture	2020	65–79	44.6	30 days	20 μg kg^−1^ of body weight of fentanyl with 0.75 bupivacaine	Fentanyl 3.5 mg kg^−1^ and propofol 1.5 mg kg^−1^	281	127	154
Tang,^41^ 2021	China	Unilateral hip fracture	2019	>65	32.7	30 days	0.25% hypobaric ropivacaine	Propofol (1–1.5 mg kg^−1^), sufentanil (0.1–0.2 μg kg^−1^), and cisatracurium besilate (0.2 mg kg^−1^)	110	55	55
Li,^24^ 2022	China	Fragility fracture (femoral neck, femoral head, intertrochanteric or subtrochanteric)	2014–8	>65	26.8	30 days	At discretion of anaesthetist in charge. No sedation given. Trial protocol advised plexus block.	At discretion of anaesthetist in charge. Induction with i.v. agents and maintenance with either i.v. or volatile agents. No sedation given. Trial protocol advised plexus block.	950	476	474

### Core outcome set

#### Delirium

**Fig 2:**
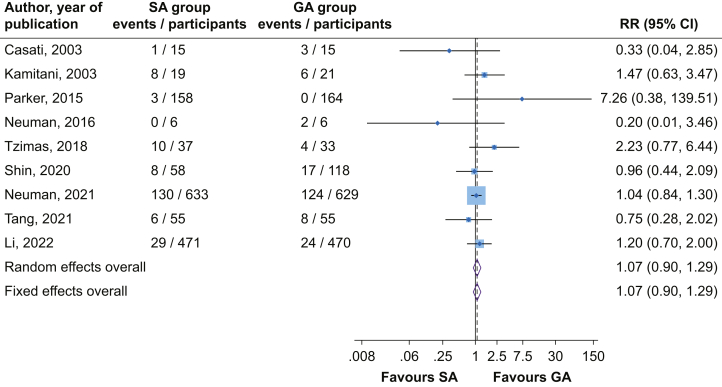
Risk of delirium comparing spinal *vs* general anaesthesia. CI, confidence interval (bars); GA, general anaesthesia; RR, relative risk; SA, spinal anaesthesia.

In pooled analysis of nine studies, there was no significant difference in the risk of delirium comparing SA *vs* GA (RR=1.07; 95% CI, 0.90–1.29), with no evidence of significant heterogeneity between contributing studies (*I*^2^=0%; 95% CI, 0–65%; *P*=0.51) (Fig 2). On exclusion of the RAGA trial which randomised spinal plus epidural anaesthesia *vs* GA,^24^ the result was similar (RR=1.06; 95% CI, 0.87–1.30). In subgroup analyses, the risk of delirium did not vary significantly between SA and GA across several study-level characteristics including location, baseline age, follow-up time, size of study, and risk of bias domains such as random sequence generation and allocation concealment (Supplementary material 4). A single study did not provide the actual number of patients who developed postoperative delirium but reported no significant difference in this outcome between the anaesthetic techniques.^52^ There were no significant differences in mini-mental state examination (MMSE) scores between both anaesthetic groups at days 1, 5, 7, and 30 postoperatively (Supplementary material 5).

#### Hypotension

**Fig 3:**
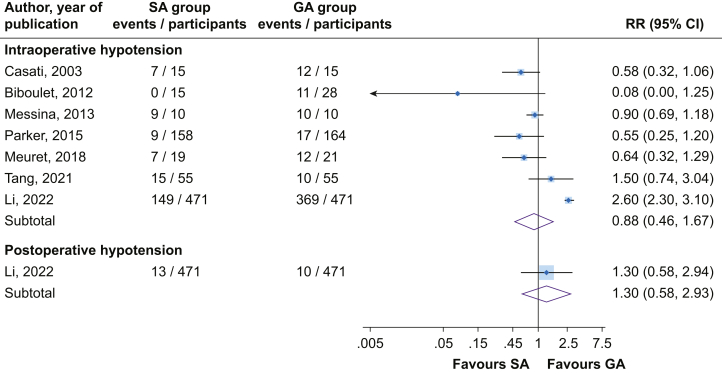
Risk of hypotension comparing spinal *vs* general anaesthesia. CI, confidence interval (bars); GA, general anaesthesia; RR, relative risk; SA, spinal anaesthesia.

There was no significant difference in the risk of intraoperative hypotension (seven studies) comparing SA *vs* GA (RR=0.88; 95% CI, 0.46–1.67), with significant heterogeneity between contributing studies (*I*^2^=93%; 95% CI, 88–96%; *P*<0.001) (Fig 3). On exclusion of the RAGA trial,^24^ the lack of significant difference persisted: RR=0.76 (95% CI, 0.54–1.09). Results from a single which report showed no significant difference in the risk of postoperative hypotension comparing SA *vs* GA (Fig 3). A number of trials which reported blood pressure changes preoperatively after induction, intraoperatively and postoperatively, did not find significant differences between SA and GA.^43^, ^44^, ^45^, ^46^

#### Mortality

**Fig 4:**
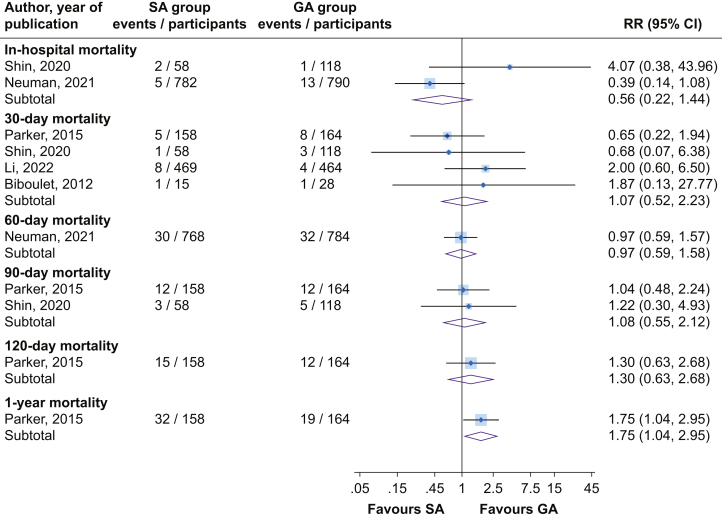
Risk of mortality comparing spinal *vs* general anaesthesia. CI, confidence interval (bars); GA, general anaesthesia; RR, relative risk; SA, spinal anaesthesia.

There was no significant difference in the risk of 30-day mortality (four studies) comparing SA *vs* GA (RR=1.07; 95% CI, 0.52–2.23), with no evidence of significant heterogeneity between contributing studies (*I*^2^=0%; 95% CI, 0–85%; *P*=0.54) (Fig 4). The lack of a significant difference remained on exclusion of the RAGA trial^24^: RR=0.74 (95% CI, 0.30–1.86). Comparing SA with GA, there were no significant differences in in-hospital, 60-day, 90-day, and 120-day mortality (Fig 4). Results from a single report showed SA increased the risk of 1-yr mortality compared with GA (Fig 4).

#### Acute coronary syndrome

**Fig 5:**
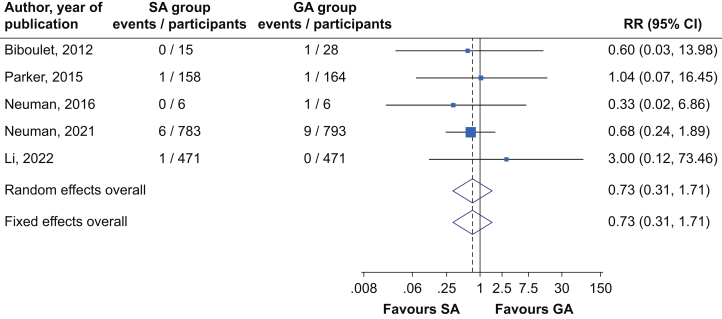
Risk of acute coronary syndrome comparing spinal *vs* general anaesthesia. CI, confidence interval (bars); GA, general anaesthesia; RR, relative risk; SA, spinal anaesthesia.

Comparing SA with GA, there was no significant difference in the risk of acute coronary syndrome (five studies): RR=0.73 (95% CI, 0.31–1.71), with no evidence of significant heterogeneity between contributing studies (*I*^2^=0%; 95% CI, 0–79%; *P*=0.89) (Fig 5). The risk was similar on exclusion of the RAGA trial^24^: RR=0.66 (95% CI, 0.27–1.59).

#### Pneumonia

In pooled analysis of three studies, there was no significant difference in the risk of pneumonia comparing SA *vs* GA (RR=0.53; 95% CI, 0.25–1.10), with no evidence of significant heterogeneity between contributing studies (*I*^2^=0%; 95% CI, 0–90%; *P*=0.92) (Supplementary material 6). The risk was similar on exclusion of the RAGA trial^24^: RR=0.54 (95% CI, 0.25–1.15).

#### Acute kidney injury

In pooled analysis of 2 studies, SA reduced the risk of AKI compared with GA: RR=0.59 (95% CI, 0.39–0.89) (Supplementary material 7).

#### Being out of bed at day 1 postoperatively

No study specifically evaluated the outcome of ‘Being out of bed at day 1 post-operatively’. However, using hospital length of stay and ward stay as proxies of ‘Being out of bed at day 1 post-operatively’, there were no significant differences between the two anaesthetic groups: mean differences (95% CIs) of 0.90 (95% CI, –1.44 to 3.24) and –0.14 (95% CI, –0.71 to 0.43) days for orthopaedic ward stay and hospital length of stay, respectively (Supplementary material 8).

#### Pain

Pain was reported using a variety of measures/scores and could not be pooled. Tzimas and colleagues^50^ reported no significant difference in pain intensity between SA and GA preoperatively (*P*=0.93) and postoperatively (*P*=0.19) using the VAS score. Casati and colleagues^44^ reported that pain relief was better controlled in the SA group immediately after discharge from the PACU, but was similar between the two groups 3 h after. Tang and colleagues^23^ reported no significant difference in mild-to-moderate pain between the two groups. The worst pain score was not significantly different between the two groups as reported by Li and colleagues.^24^

#### Time from injury to surgery

The pooled results of two studies showed no significant difference in time from injury to surgery as assessed by ‘Delay in surgery’ comparing SA with GA: mean difference (95% CI) of 0.00 (95% CI, –0.30 to 0.31) days (Supplementary material 9).

#### Orthogeriatric input

No study reported on orthogeriatric input.

#### Quality of life

No study reported on QOL. Using patient satisfaction as a surrogate of QOL, results of a single report showed no significant difference comparing SA with GA (Supplementary material 10).

#### Mobility status

Results of a single report showed no significant difference in mobility status comparing SA with GA as assessed by the following measures: Inability to walk without human assistance among survivors at 60 days; Worsened walking ability; and 12-item World Health Organization Disability Schedule 2.0 (WHODAS 2.0) (Supplementary materials 11 and 12).

#### Return to preoperative residence

Results of a single report showed no significant difference in return to preoperative residence comparing SA with GA^49^: RR=0.99 (95% CI, 0.92–1.06). In another report,^23^ SA reduced the deterioration of ADL score (an indirect marker of patient's function and independence) 30 days postoperatively.

### GRADE summary of findings

The GRADE working group recommends up to seven patient-important outcomes to be listed in the ‘summary of findings’ tables in systematic reviews.^34^ We selected the following outcomes for assessment based on their reported frequency given that all the outcomes assessed were considered to be equally important: delirium, intraoperative hypotension, acute coronary syndrome, 30-day mortality, in-hospital mortality, pneumonia, and AKI. GRADE quality of the evidence ranged from high to very low (Supplementary material 13).

## Discussion

Except for a reduced risk of AKI comparing SA with GA, this systematic review and meta-analysis of contemporary RCTs found no significant differences in the consensus-based set of core outcomes and outcomes defined as important by PPI initiatives between the two anaesthetic techniques. Most studies reported one to three outcomes from the COS with the most commonly reported outcomes being mortality, delirium, hypotension, and acute coronary syndrome; only three studies reported on six to seven outcomes from the COS. No study reported on the core outcomes of orthogeriatric input or QOL and the findings on PPI outcomes were mostly based on single reports. The GRADE quality of evidence for the seven most frequently reported outcomes ranged from high to very low.

Several reviews of RCTs have previously reported on the comparative effectiveness and safety of SA *vs* GA in patients undergoing hip fracture surgery. In addition to addressing some limitations of these reviews such as inclusion of studies that employed anaesthetic drugs not considered usual contemporary clinical practice^12^ and selective reporting of outcomes,^16^ our systematic review and meta-analysis utilised several new approaches previously not reported. We included only contemporary RCTs (published from 2000 to date) and compared the two anaesthetic techniques using a consensus-based set of 10 core outcomes and those defined as important by PPI initiatives. Although our methodology cannot be directly compared with other published relevant reviews, some of our findings can be compared with these reviews. In a Cochrane review involving the comparison of neuraxial block (spinal, epidural, or the combination) *vs* GA, there were no differences between the two anaesthetic techniques for 30-day mortality, pneumonia, and MI.^12^ Zheng and colleagues,^15^ in their analysis of 9 RCTs comparing neuraxial anaesthesia (spinal or epidural) with GA, demonstrated no significant differences in the risk of 30-day mortality, delirium, acute MI, and pneumonia. Urwin and colleagues,^53^ in pooled analysis of 15 RCTs published before the year 2000, showed no significant differences in a comprehensive list of outcomes between regional and GA except for a reduced risk of 30-day mortality with regional anaesthesia. In addition to reporting on outcomes that have not been previously considered in past reviews such as QOL, mobility status, and return to preoperative residence, we have also shown that SA reduced the risk of AKI, albeit based on pooled analysis of only two studies. Furthermore, a subgroup analysis showed that the risk of delirium comparing the two anaesthetic techniques was not modified by location, age, time of ascertainment of event, or other aspects of the study design.

With regard to the panel of outcomes assessed, SA appears to be equivalent to GA in terms of effectiveness and safety. The lack of significant differences across outcomes between the two anaesthetic techniques has been demonstrated in previous reviews.^12^, ^13^, ^14^, ^15^ This lack of difference may not equate to equivalence. The substantial variation and heterogeneity across the studies with respect to anaesthetic regimen, dosages, depth and sedation, patient characteristics and their risk profile, anaesthetic and surgical expertise, and outcomes means effective head-to-head comparisons are not possible.

Large multicentre trials with robust designs and methodology could help address any differences between the two anaesthetic techniques. Two large multicentre RCTs (RAGA and REGAIN)^24^^,^^25^ were designed to assess clinical effectiveness of regional anaesthesia and GA. Whereas REGAIN compared only spinal anaesthesia with GA, the RAGA trial compared three neuraxial techniques (spinal, epidural, or combined spinal epidural) with GA. Their results provided crucial randomised data but some uncertainties between the two anaesthetic techniques remained unsolved. The REGAIN trial concluded that regional anaesthesia did not provide better outcomes after hip fracture surgery compared with GA. This pragmatic study examined patient-centred outcomes including ambulation, new onset delirium, and mortality, but the study may have lacked sufficient power to detect difference because of a lower-than-expected incidence of the primary outcome. Their primary outcome was a composite measure of mortality and level of dependence at 60 days after randomisation which may preclude the ability to detect significant differences in other clinically relevant outcomes such as development of AKI. There was a significant rate of crossover from spinal into the GA group and the use of sedation in the SA group was allowed. Although this pragmatic approach and crossover may reflect real practice, it may have affected the study's ability to discern the effect of these different anaesthesia regimes. In comparison, participants in the neuraxial anaesthesia group in the RAGA study did not receive additional sedation but the study did not find a difference in reported rates of postoperative delirium. Mortality and rates of postoperative delirium were substantially lower than in previous studies, and it is possible that differences in the patient population in China mean that results are less generalisable to other countries. Anaesthetic practice in the RAGA study was notably more variable with the majority of patients in the neuraxial group receiving combined spinal and epidural anaesthesia, which is not commonly used in other countries. There were also a proportion of patients who underwent both neuraxial anaesthesia and GA but were included in the GA group for analysis. Crucially, the participants in both trials were younger than the average age reported by national registries such as the UK National Hip Fracture Database (NHFD) (average age at randomisation was 77 yr in both trials *vs* 83.5 yr based on National Hip Fracture Database (NHFD) data).^54^ The participants in RAGA mostly had American Society of Anesthesiologists (ASA) grade 2, thus suggesting a less comorbid state at baseline which may have affected their results as preoperative frailty has been shown to be a strong predictor for postoperative cognitive decline and morbidity.^55^ Neither RAGA nor REGAIN accounted for other important factors in perioperative care which may affect key outcomes such as time to surgery and strategies for postoperative analgesia. Furthermore, the trials used different screening tests for delirium; the heterogeneity between assessment methods limits their direct comparison.

It is uncertain if the ongoing Improve Hip Fracture Outcome In The Elderly Patient (iHOPE) trial,^56^ which intends to randomise 1032 patients with hip fracture to receive SA (*n*=516) or GA (*n*=516), will provide definite conclusions on which anaesthetic technique is superior. However, any observed effects would have to be large to lead to a change in the conclusions of this evidence synthesis given the sample sizes and confidence intervals.

The overall evidence suggests that using either SA or GA in patients undergoing hip fracture surgery did not impact on patient outcomes. Given the uncertainty regarding which anaesthetic technique is superior, the Association of Anaesthetists advocates for a shift in focus away from demonstrating superiority but rather to optimise anaesthetic delivery in a patient-centred manner.^57^ The clinical decision to use a particular anaesthetic is a complex process and should be based on the patient's risk profile and preference, input of the multidisciplinary team and the expertise of the anaesthetist. Future clinical trials should take into consideration the consensus-based COS and PPI outcomes. The current lack of outcomes important to patients (such as preoperative residence, QOL, and mobility status) in RCTs is concerning. Although these have been recognised as important by others, and are used as key performance indicators by the NHFD for England, Wales, and Northern Ireland to assess the quality of care provided,^5^ it is vital these outcome measures are included in future RCTs, which will help benefit both patients and clinicians in guiding the choice of anaesthetic for hip fracture surgery.

### Strengths and limitations

In comparison with previous meta-analyses, this is the first meta-analysis of RCTs to evaluate the clinical effectiveness of SA and GA using a consensus-based set of 10 core outcomes developed for evaluating the effects of anaesthesia in hip fracture surgery in addition to relevant PPI outcomes. Other strengths of the current review are (1) the addition of several new RCTs including the two most recent large trials (RAGA and REGAIN), which provided enhanced power to evaluate key outcomes; (2) the utilisation of several meta-analytic approaches including ensuring consistency to enhance pooling most of the data and quantification of heterogeneity; and (3) detailed assessment of the risk of bias of included trials and quality of the evidence using the Cochrane risk of bias and GRADE tools, respectively.

There were several limitations, which were mostly inherent and not related to our methodology. There were varied reporting in outcome definitions, time points, and assessments among eligible studies, with selective reporting of outcomes; none of the studies reported the full list of 10 core outcomes or PPI outcomes. Anaesthesia regimens and dosages also varied across trials. Furthermore, findings on outcomes defined as important by PPI initiatives were all based on single reports. The limited number of studies for the majority of outcomes precluded effective comparisons, exploration of heterogeneity, and assessment of small study effects. Stratified analyses could not be conducted for subgroups such as sex differences and use of sedation and nerve blocks in conjunction with anaesthesia, as prespecified in our registered protocol because of limited data reported by the trials. Finally, in our assessment of the risk of bias, most trials had a high risk of bias in at least one domain. Owing to lack of detailed reporting, risk of bias was rated as unclear in several domains.

### Conclusions

Using a consensus-based COS and PPI outcomes, high-to very low-quality interventional evidence suggests that SA is not significantly different to GA in patients undergoing hip fracture surgery for most outcomes assessed. However, limited evidence suggests SA reduces the risk of AKI compared with GA. Most studies reported on only one to three outcomes from the COS, and only few studies reported outcomes important to patients (including preoperative residence, QOL, and mobility status). These outcomes should be incorporated into future RCTs.

## Authors' contributions

Study concept: SKK, JY, MRW, GSM

Study design: all authors

Data collection and analysis: SKK, PBH, ST

Data interpretation: all authors

Drafting of the manuscript: SKK and GSM

Approval of the final report: interpretation

All authors agree to be accountable for all aspects of the work in ensuring that questions related to the accuracy or integrity of any part of the work are appropriately investigated and resolved.

Guarantor for this study and final responsibility for manuscript submission: GSM

## Ethics approval

Research ethics committee approval was not required as this was a systematic review of previously published studies.

## Data sharing

The study is based on data from previously published studies and is available online to all.

## Declarations of interest

JY was a co-investigator for RAGA trial. All authors declare: funding was received for the submitted work; no financial relationships with any organisations that might have an interest in the submitted work in the previous 3 yr, no other relationships or activities that could appear to have influenced the submitted work.

## Funding

Academy of Medical Sciences, the Wellcome Trust, the Medical Research Council, the British Heart Foundation, Versus Arthritis, Diabetes UK, the British Thoracic Society (Helen and Andrew Douglas bequest), and the Association of Physicians of Great Britain and Ireland (SGL023∖1021 to GSM). NIHR Biomedical Research Centre at University Hospitals Bristol NHS Foundation Trust and the University of Bristol.
