# Supplementary material for: Clinical effectiveness and safety of spinal anaesthesia compared with general anaesthesia in patients undergoing hip fracture surgery using a consensus-based core outcome set and patient-and public-informed outcomes: a systematic review and meta-analysis of randomised controlled trials
Source: Br J Anaesth. 2022 Sep 28;129(5):788–800. doi: 10.1016/j.bja.2022.07.031 (PMC9642835; doi:10.1016/j.bja.2022.07.031)
Supplement: Multimedia component 1 [file mmc1.docx]

**Supplementary Material**

| **Supplementary Material 1** | PRISMA checklist |
| --- | --- |
| **Supplementary Material 2** | Literature search strategy |
| **Supplementary Material 3** | Risk of bias assessment |
| **Supplementary Material 4** | Risk of delirium comparing spinal versus general anaesthesia, according to study-level characteristics |
| **Supplementary Material 5** | Spinal versus general anaesthesia and mini-mental state examination scores |
| **Supplementary Material 6** | Spinal versus general anaesthesia and pneumonia |
| **Supplementary Material 7** | Spinal versus general anaesthesia and acute kidney injury |
| **Supplementary Material 8** | Spinal versus general anaesthesia and “Being out of bed at day 1 postoperatively” |
| **Supplementary Material 9** | Spinal versus general anaesthesia and “Time from injury to surgery” |
| **Supplementary Material 10** | Spinal versus general anaesthesia and Quality of life |
| **Supplementary Material 11** | Spinal versus general anaesthesia and Mobility status as a binary outcome |
| **Supplementary Material 12** | Spinal versus general anaesthesia and Mobility status as a continuous outcome |
| **Supplementary Material 13** | GRADE summary of findings |

**Supplementary Material 1.** PRISMA checklist

| **Section/topic** | **Item No** | **Checklist item** | **Reported on page No** |
| --- | --- | --- | --- |
| **Title** | | | |
| Title | 1 | Identify the report as a systematic review, meta-analysis, or both | 1 |
| **Abstract** | | | |
| Structured summary | 2 | Provide a structured summary including, as applicable, background, objectives, data sources, study eligibility criteria, participants, interventions, study appraisal and synthesis methods, results, limitations, conclusions and implications of key findings, systematic review registration number | 2 |
| **Introduction** | | | |
| Rationale | 3 | Describe the rationale for the review in the context of what is already known | Introduction |
| Objectives | 4 | Provide an explicit statement of questions being addressed with reference to participants, interventions, comparisons, outcomes, and study design (PICOS) | Introduction |
| **Methods** | | | |
| Protocol and registration | 5 | Indicate if a review protocol exists, if and where it can be accessed (such as web address), and, if available, provide registration information including registration number | Methods |
| Eligibility criteria | 6 | Specify study characteristics (such as PICOS, length of follow-up) and report characteristics (such as years considered, language, publication status) used as criteria for eligibility, giving rationale | Methods |
| Information sources | 7 | Describe all information sources (such as databases with dates of coverage, contact with study authors to identify additional studies) in the search and date last searched | Methods |
| Search | 8 | Present full electronic search strategy for at least one database, including any limits used, such that it could be repeated | Supplementary Material 2 |
| Study selection | 9 | State the process for selecting studies (that is, screening, eligibility, included in systematic review, and, if applicable, included in the meta-analysis) | Methods |
| Data collection process | 10 | Describe method of data extraction from reports (such as piloted forms, independently, in duplicate) and any processes for obtaining and confirming data from investigators | Methods |
| Data items | 11 | List and define all variables for which data were sought (such as PICOS, funding sources) and any assumptions and simplifications made | Methods |
| Risk of bias in individual studies | 12 | Describe methods used for assessing risk of bias of individual studies (including specification of whether this was done at the study or outcome level), and how this information is to be used in any data synthesis | Methods |
| Summary measures | 13 | State the principal summary measures (such as risk ratio, difference in means). | Methods |
| Synthesis of results | 14 | Describe the methods of handling data and combining results of studies, if done, including measures of consistency (such as I^2^ statistic) for each meta-analysis | Methods |
| Risk of bias across studies | 15 | Specify any assessment of risk of bias that may affect the cumulative evidence (such as publication bias, selective reporting within studies) | Methods |
| Additional analyses | 16 | Describe methods of additional analyses (such as sensitivity or subgroup analyses, meta-regression), if done, indicating which were pre-specified | Methods |
| **Results** | | | |
| Study selection | 17 | Give numbers of studies screened, assessed for eligibility, and included in the review, with reasons for exclusions at each stage, ideally with a flow diagram | Results and Figure 1 |
| Study characteristics | 18 | For each study, present characteristics for which data were extracted (such as study size, PICOS, follow-up period) and provide the citations | Results; Table 1; |
| Risk of bias within studies | 19 | Present data on risk of bias of each study and, if available, any outcome-level assessment (see item 12). | Results; Table 1; |
| Results of individual studies | 20 | For all outcomes considered (benefits or harms), present for each study (a) simple summary data for each intervention group and (b) effect estimates and confidence intervals, ideally with a forest plot | Results; Figures 2-5; Supplementary Material 4-12 |
| Synthesis of results | 21 | Present results of each meta-analysis done, including confidence intervals and measures of consistency | Results; Figures 2-5; Supplementary Material 4-12 |
| Risk of bias across studies | 22 | Present results of any assessment of risk of bias across studies (see item 15) | Supplementary Material 3 |
| Additional analysis | 23 | Give results of additional analyses, if done (such as sensitivity or subgroup analyses, meta-regression) (see item 16) | Supplementary Material 4 |
| **Discussion** | | | |
| Summary of evidence | 24 | Summarise the main findings including the strength of evidence for each main outcome; consider their relevance to key groups (such as health care providers, users, and policy makers) | Discussion |
| Limitations | 25 | Discuss limitations at study and outcome level (such as risk of bias), and at review level (such as incomplete retrieval of identified research, reporting bias) | Discussion |
| Conclusions | 26 | Provide a general interpretation of the results in the context of other evidence, and implications for future research | Discussion |
| **Funding** | | | |
| Funding | 27 | Describe sources of funding for the systematic review and other support (such as supply of data) and role of funders for the systematic review | After discussion |

**Supplementary Material 2.** Literature search strategy

Relevant studies, published from 2000 to 10 February 2022 (date last searched), were identified through electronic searches using MEDLINE, Embase, and Cochrane databases. Electronic searches were supplemented by scanning reference lists of articles identified for all relevant studies (including review articles) and Web of Science citation checking

| 1 exp Hip Fractures/ (26005)  2 exp Femoral Fractures/ or femur fracture.mp. (42761)  3 femoral neck.mp. or exp Femur Neck/ (26084)  4 trochant*.mp. (9254)  5 intracapsular*.mp. (3488)  6 extracapsular*.mp. (6852)  7 regional an?esthesia.mp. (9572)  8 exp Anesthesia, Spinal/ (12583)  9 neuraxial an?esthesia.mp. (1121)  10 exp Anesthesia, Epidural/ (13849)  11 exp Anesthesia, General/ (60072)  12 an?esthesia.mp. (298977)  13 ("clinical trial" or "clinical trial, phase i" or "clinical trial, phase ii" or clinical trial, phase iii or clinical trial, phase iv or controlled clinical trial or "multicenter study" or "randomized controlled trial").pt. or double-blind method/ or clinical trials as topic/ or clinical trials, phase i as topic/ or clinical trials, phase ii as topic/ or clinical trials, phase iii as topic/ or clinical trials, phase iv as topic/ or controlled clinical trials as topic/ or randomized controlled trials as topic/ or early termination of clinical trials as topic/ or multicenter studies as topic/ or ((randomi?ed adj7 trial*) or (controlled adj3 trial*) or (clinical adj2 trial*) or ((single or doubl* or tripl* or treb*) and (blind* or mask*))).ti,ab,kw. or ("4 arm" or "four arm").ti,ab,kw. (1755030)  14 1 or 2 or 3 or 4 or 5 or 6 (71254)  15 7 or 8 or 9 or 10 or 11 or 12 (298977)  16 13 and 14 and 15 (298)  17 limit 16 to (humans and yr="2000 -Current") (211)  17 limit 16 to humans (3890)  Each part was specifically translated for searching the other databases (Embase and Cochrane databases) |
| --- |

**Supplementary Material 3.** Risk of bias assessment for randomised controlled trials

**Supplementary Material 4.** Risk of delirium comparing spinal versus general anaesthesia, according to study-level characteristics

CI, confidence intervals (bars); GA, general anaesthesia; RR, relative risk; SA, spinal anaesthesia *, p-value for meta-regression

**Supplementary Material 5.** Spinal versus general anaesthesia and mini-mental state examination scores

CI, confidence intervals (bars); GA, general anaesthesia; MMSE, mini-mental state examination; SA, spinal anaesthesia

**Supplementary Material 6.** Spinal versus general anaesthesia and pneumonia

CI, confidence intervals (bars); GA, general anaesthesia; RR, relative risk; SA, spinal anaesthesia

**Supplementary Material 7.** Spinal versus general anaesthesia and acute kidney injury

CI, confidence intervals (bars); GA, general anaesthesia; RR, relative risk; SA, spinal anaesthesia

**Supplementary Material 8.** Spinal versus general anaesthesia and “Being out of bed at day 1 postoperatively”

CI, confidence intervals (bars); GA, general anaesthesia; SA, spinal anaesthesia

**Supplementary Material 9.** Spinal versus general anaesthesia and “Time from injury to surgery”

CI, confidence intervals (bars); GA, general anaesthesia; SA, spinal anaesthesia

**Supplementary Material 10.** Spinal versus general anaesthesia and Quality of life

CI, confidence intervals (bars); GA, general anaesthesia; RR, relative risk; SA, spinal anaesthesia

**Supplementary Material 11.** Spinal versus general anaesthesia and Mobility status as a binary outcome

CI, confidence intervals (bars); GA, general anaesthesia; RR, relative risk; SA, spinal anaesthesia

**Supplementary Material 12.** Spinal versus general anaesthesia and Mobility status as a continuous outcome

CI, confidence intervals (bars); GA, general anaesthesia; SA, spinal anaesthesia

**Supplementary Material 13.** GRADE summary of findings

| **Outcomes** | **№ of participants (studies) Follow-up** | **Certainty of the evidence (GRADE)** | **Relative effect (95% CI)** | **Anticipated absolute effects** | |
| --- | --- | --- | --- | --- | --- |
|  |  |  |  | **Risk with General anaesthesia** | **Risk difference with Spinal anaesthesia** |
| Delirium | 2963 (9 RCTs) | ⨁⨁⨁◯ Moderate^a^ | **RR 1.07** (0.90 to 1.29) | 124 per 1,000 | **9 more per 1,000** (12 fewer to 36 more) |
| Intra-operative hypotension | 1507 (7 RCTs) | ⨁◯◯◯ Very low^a,b^ | **RR 0.88** (0.46 to 1.67) | 577 per 1,000 | **69 fewer per 1,000** (312 fewer to 387 more) |
| Acute coronary syndrome | 2895 (5 RCTs) | ⨁⨁⨁◯ Moderate^a^ | **RR 0.73** (0.31 to 1.71) | 8 per 1,000 | **2 fewer per 1,000** (6 fewer to 6 more) |
| 30-day mortality | 1474 (4 RCTs) | ⨁⨁◯◯ Low^a,c^ | **RR 1.07** (0.52 to 2.23) | 21 per 1,000 | **1 more per 1,000** (10 fewer to 25 more) |
| In-hospital mortality | 1760 (2 RCTs) | ⨁⨁◯◯ Low^a,d^ | **RR 0.56** (0.22 to 1.44) | 15 per 1,000 | **7 fewer per 1,000** (12 fewer to 7 more) |
| Pneumonia | 2840 (3 RCTs) | ⨁⨁⨁◯ Moderate^a^ | **RR 0.53** (0.25 to 1.10) | 14 per 1,000 | **7 fewer per 1,000** (11 fewer to 1 more) |
| Acute kidney injury | 1757 (2 RCTs) | ⨁⨁⨁⨁ High | **RR 0.59** (0.39 to 0.89) | 64 per 1,000 | **26 fewer per 1,000** (39 fewer to 7 fewer) |
| ***The risk in the intervention group** (and its 95% confidence interval) is based on the assumed risk in the comparison group and the **relative effect** of the intervention (and its 95% CI).  **CI:** confidence interval; **RR:** risk ratio | | | | | |
| **GRADE Working Group grades of evidence** **High certainty:** we are very confident that the true effect lies close to that of the estimate of the effect. **Moderate certainty:** we are moderately confident in the effect estimate: the true effect is likely to be close to the estimate of the effect, but there is a possibility that it is substantially different. **Low certainty:** our confidence in the effect estimate is limited: the true effect may be substantially different from the estimate of the effect. **Very low certainty:** we have very little confidence in the effect estimate: the true effect is likely to be substantially different from the estimate of effect. | | | | | |

**Explanations**

a. High risk of bias in one or more domains

b. I-squared value of 93%.

c. Wide 95% CI (0.52-2.23)

d. I-squared value of 68.4%
